# The global research status and trends of the application of endoscopic ultrasonography in pancreatic tumors over the last decades: A bibliometric study

**DOI:** 10.3389/fonc.2022.980415

**Published:** 2022-08-12

**Authors:** Chuanchao Xia, Hua Yin, Kecheng Zhang, Zhenhuan Wang, Xiaoli Yang, Haojie Huang

**Affiliations:** ^1^ Department of Gastroenterology, First Affiliated Hospital, Naval Medical University, Shanghai, China; ^2^ Department of Gastroenterology, General Hospital of Ningxia Medical University, Yinchuan, China; ^3^ Department of Biliary Tract Surgery, Eastern Hepatobiliary Surgery Hospital, Shanghai, China; ^4^ School of Biomedical Engineering and Technology, Tianjin Medical University, Tianjin, China

**Keywords:** endoscopic ultrasonography (EUS), pancreatic tumors, bibliometric analysis, application, trend

## Abstract

**Background:**

To describe the development process and structural relationships of scientific achievements in endoscopic ultrasonography (EUS) in pancreatic tumors over the past decades and to reveal the key research topics using bibliometric analysis.

**Methods:**

All relevant publications covering the research of EUS in pancreatic tumors from 1984 to 2021 were involved through the Web of Science Core Collection. R-bibliometrix was used to conduct the bibliometric analysis, and VOSviewer software was used to explore the hot spots and networks related to this field.

**Results:**

Between 1984 and 2021, 4071 publications were involved. The number of annual publications increased from 1 to 310. The United States contributed the most publications to this field (n=1433, 35.20%), followed by Japan (n=827, 20.31%) and Germany (n=319, 7.84%). There was active cooperation between countries/regions. Gastrointestinal Endoscopy (GIE) was the most productive journal and the most influential journal. Professor Giovannini M, who produced the most publications, had a great influence on this research. The focus in this field was clarified by analyzing the top 10 citations and co-citations publications. Moreover, the analysis of the keywords showed Important topics: “Classification of pancreatic tumor disease” “Development of EUS in the diagnosis of pancreatic tumor diseases,” and “Development of EUS in the treatment of pancreatic tumor diseases.”

**Conclusion:**

For the first time, bibliometric analysis was used to gain a deep understanding of the global trends of studies investigating EUS in pancreatic tumor diseases. The EUS field is rapidly evolving, and our study may be a critical reference for clinical researchers related to this field.

## Introduction

Flexible endoscopy was created in 1911, and ultrasound (US) followed in 1956. By combining ultrasonography and endoscopy, endoscopic ultrasonography (EUS) created an entirely new dimension in imaging (1). EUS was developed in the early 1980s, it places an ultrasound probe on the tip of the endoscope to directly observe the morphological structure and lesions of the mucosal surface as well as visualize the tube wall hierarchy, lesion origin, and infiltration depth using ultrasound technology. Due to the proximity of the US transducer to the lesion and the use of a high-frequency US probe, the picture resolution was improved (2).

The pancreas is a retroperitoneal organ, and traditional imaging diagnosis of pancreatic diseases is so inaccurate that malignant tumors are typically detected late in their progression, resulting in missed opportunities for surgery. Pancreatic neoplasms include benign pancreatic neoplasms and pancreatic malignancies, among which pancreatic cancer is one of the most deadly malignancies, with a fatality rate that is roughly equivalent to its incidence (3), and its five-year survival rate is still low. The aggressive biology, inefficient therapy, and advanced stages at the time of diagnosis all contribute to the disease’s poor prognosis (4). As a result, detecting precursor lesions, early diagnosis, or effective treatment strategy may be a viable way for extending survival. Using EUS, images of the pancreas can be captured through the esophagus, stomach, and duodenum without being obstructed by gas, fat, or bone, and EUS also is allowed for tissue collection, making it the most widely used screening technology for early pancreatic tumors diagnosis (5, 6).

Lots of studies have shown that EUS and its related techniques, such as EUS elastography (EUS-EG), EUS-guided fine needle aspiration (EUS-FNA) and contrast-enhanced EUS (CE-EUS) are now considered to be the most sensitive imaging modality for the clinical diagnosis of pancreatic lesions, along with the detection of small cancers, the variability diagnosis of pancreatic solid or cystic lesions, and the staging of pancreatic lesions. Since the first clinical application of EUS in the 1980s, more than 30 years of development have changed various fields of gastroenterology. EUS has become a vital diagnostic and therapy tool, with more and more diagnostic and treatment recommendations recommending its use for inspection and treatment, particularly in pancreatic and gastrointestinal diseases (7).

It is expected that if we can analyze the recent research status of the application of EUS in pancreatic tumor diseases found in the literature that is presently available and explore the current research challenges and future research hotspots, we will be able to provide references for researchers interested in pancreatic tumors. Bibliometrics is the tool for achieving the aforementioned objectives. Using mathematical and statistical techniques, bibliometrics is the multidisciplinary study of all knowledge bearers, such as books, magazines, and other publications (8). It is a broad knowledge base that integrates philology, mathematics, and statistics with a focus on quantification. In addition to evaluating the contributions of various nations, institutions, journals, and scholars, bibliometric analysis can also describe a particular research area, foresee particular trends, and identify potential future research hotspots, all of which have a significant impact on disease prevention and treatment (9).

However, no study on the analysis of EUS in pancreatic tumors from a literature perspective exists, thus for the first time, we were able to acquire a comprehensive understanding of the global trends of studies examining EUS in pancreatic disease from multiple perspectives. Using the Web of Science (WOS), the goal of this study is to explore the content of the research literature on the application of EUS in pancreatic tumors. To provide a resource for clinical care and academic research in this area, we use the bibliometric strategies to conduct an extensive analysis of this field’s research status throughout the previous decades, identify research trends, and predict probable future research hotspots.

## Methods

### Data source and collection

**Figure 1:**
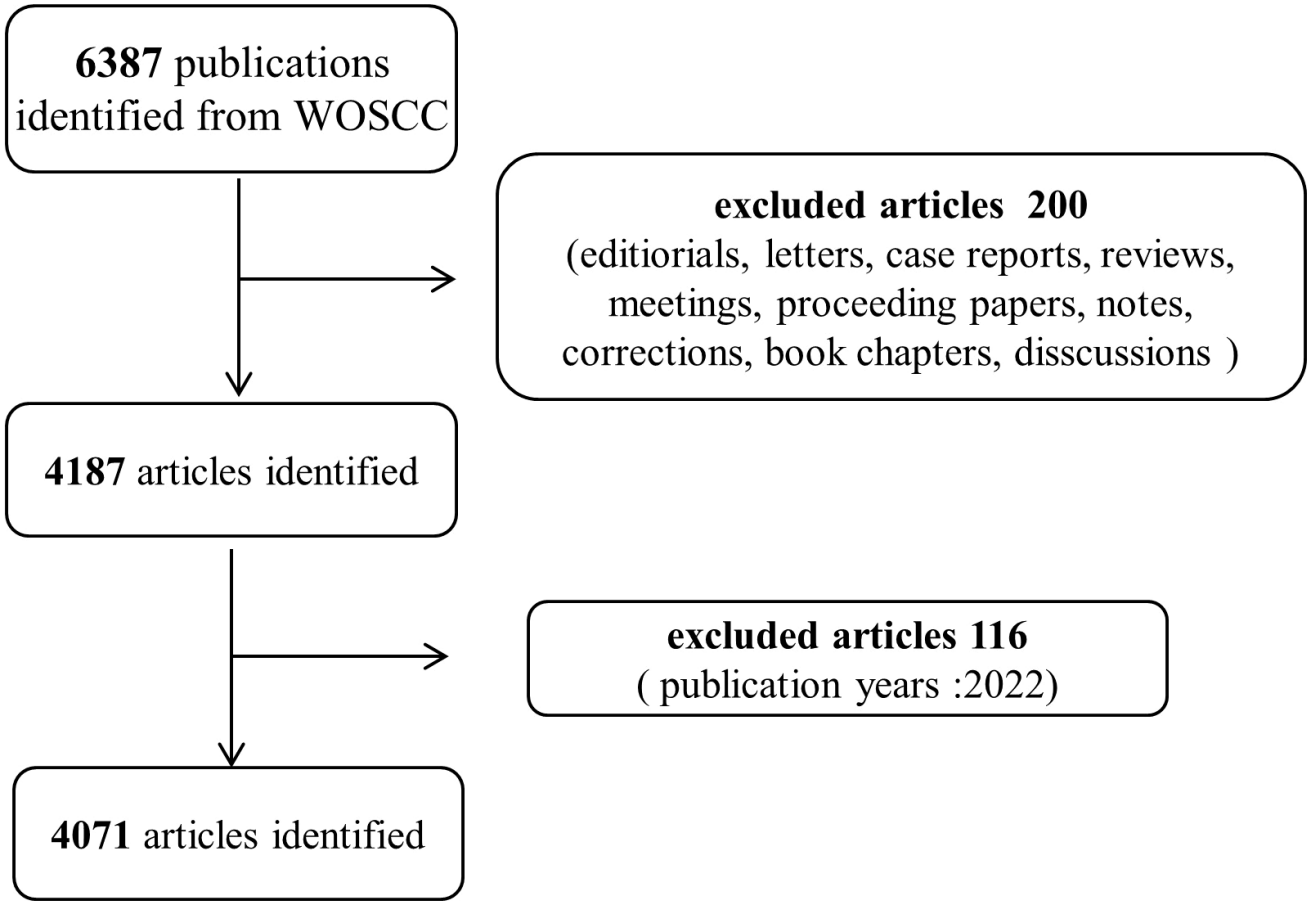
Flow diagram of literature selection and screening in this study.

A comprehensive collection was conducted of all of the publications from the Web of Science Core Collection (WOSCC) from 1984 to 2021 with the following retrieval strategies: [(endoscopic ultrasound) OR (endoscopic ultrasonography)] OR (EUS)] and [(Pancreatic cancer) or (PDAC) or (pancreatic tumor) or (pancreatic neoplasms) or (pancreatic cystic tumors)], and only “articles” was included. To avoid citation variations caused by frequent database updates, all related records were downloaded on May 4, 2022, and imported into relevant bibliometric tools for further analysis. All primary searches were independently performed by two authors (Xia and zhang), and their agreement showed considerable accordance. **Figure 1** depicts the complete procedure of literature selection and screening.

### Statistical analysis

We attempted to compile “The WOSCC Literature Analysis Report” to summarize publication features such as authors, nations, journals, institution status, annual publications, H index, and relations between authors or countries. By reviewing the most recent JCR (Journal Citation Reports) issue, a crucial tool for determining the academic effect of research, we obtained the most recent impact factor (IF) of the pertinent journals. The H-index is a statistic for evaluating an individual’s contribution to scientific research (10). and the H-index, which reflects the academic influence of researchers or countries/regions, can be found on WOS.

We evaluated the number of publications and growth trends in various nations/areas using the bibliometrics online analytical platform. VOSviewer software was used to visualize keyword networks generated from related studies, allowing co-occurrence analysis to categorize terms into different clusters. The average year of appearance definition, which we used to gauge the relative novelty of terms, also colored each keyword when it first appeared.

## Results

### Annual output

**Figure 2:**
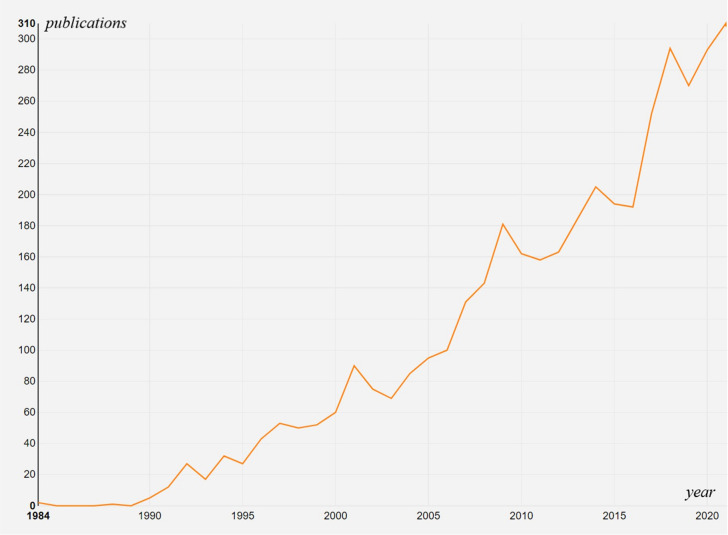
Times Cited and Publications Over Time.

According to the retrieval strategy, 6387 publications were included initially. 200 were excluded for unmatched document types, and 116 were excluded for published in 2022. In the end, 4071 “articles” were selected to perform analysis. The total number of publications worldwide was analyzed and an increasing trend in the total publishing has been demonstrated (from 1 in 1984 to 310 in 2021). The number of publications showed two stages: less before 2000 and more after 2000. The number of annual publications was more than 60 after 2000, more than 200 after 2014, and more than 300 in 2021 (the number was nearly 300 in 2018, and 2020). The annual publication outputs of the application of EUS in the pancreatic tumors field are shown in **Figure 2**. English was the most popular language in this subject, making up 94.92 percent of the total. German was the most prevalent non-English language, making up 2.11 percent of the total, followed by French (1.65%), Spanish (0.93%), and others such as Hungarian, Italian, Chinese, et al. are under 0.01%.

### Country/region analysis

**Figure 3:**
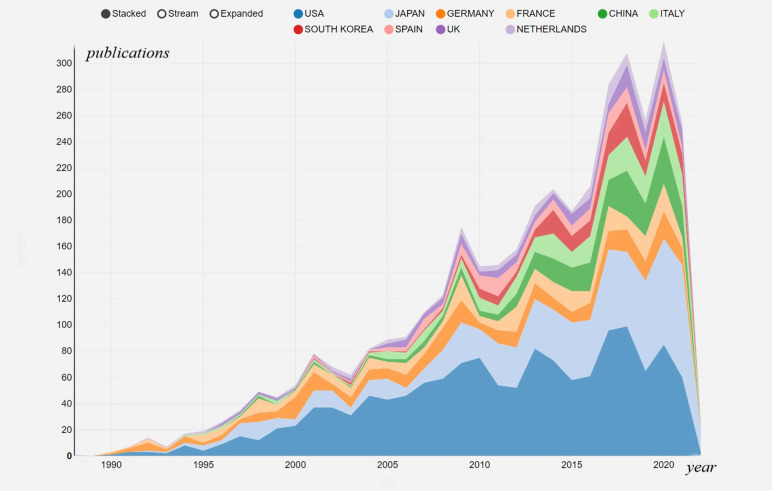
The contributions of different countries/regions to the research field.

A total of 80 countries or regions participated in publishing articles on this research over the past few decades. The United States published the highest number of articles (n=1433, 35.20%), followed by Japan (n=827, 20.31%), Germany (n=319, 7.84%), France (n=280, 6.88%), Italy (n=264, 6.46%), China (n=225, 5.53%), South Korea (n=167, 4.10%), Spain (n=145, 3.56%), England (n=131, 3.22%), Netherlands (n=117, 2.87%). The top 10 countries/regions were showed in **Figure 3**. The outcomes of the cited frequency report from the WOSCC indicated that 4071 articles connected to this field were referenced 119,150 times since 2001, and 90,462 times without self-citation. The average citation frequency per literature was 29.27, and the H-index was 141. The United States had the highest citation frequency (58,351 times, 51,569 times without self-citation). With an H-index of 116, the average number of citations per piece of literature was 49.72. With an H-index of 65, Japan’s publications were mentioned 18,404 times (16,735 times without self-citation), placing it second among all nations.

**Figure 4:**
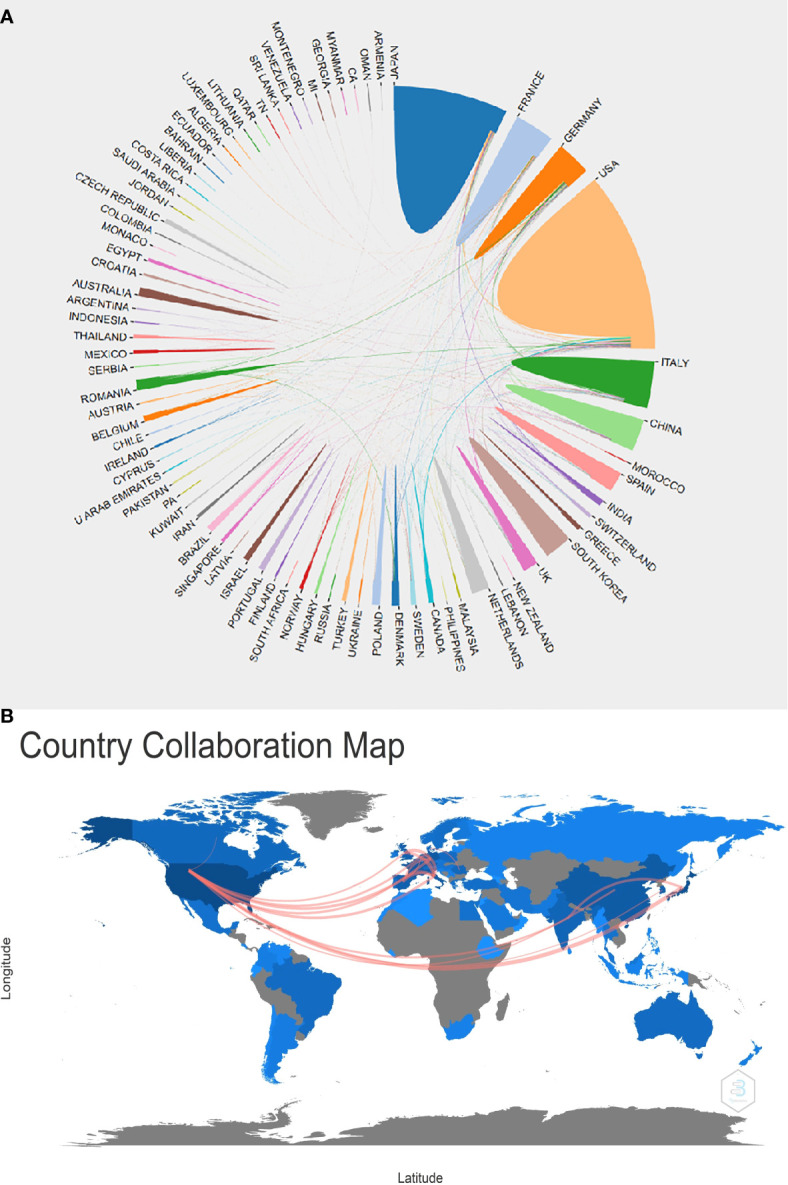
Country/region analysis. **(A)**The cooperation of countries/regions in this field from 1984 to 2021. **(B)** Country collaboration map in this field from 1984 to 2021.

We mapped the cooperation of countries and regions to have a better understanding of the extent of collaboration between them globally. Not only did the United States have the highest number of publications, but also had the closest international collaboration, which was shown by the U.S.’s prominent position in the co-occurrence network (**Figure 4A**). Active partnerships between nations and areas were shown on the visualization map; for instance, the USA had tight ties to Japan, Germany, France, and Italy (**Figure 4B**).

### Journal analysis

**Figure 5:**
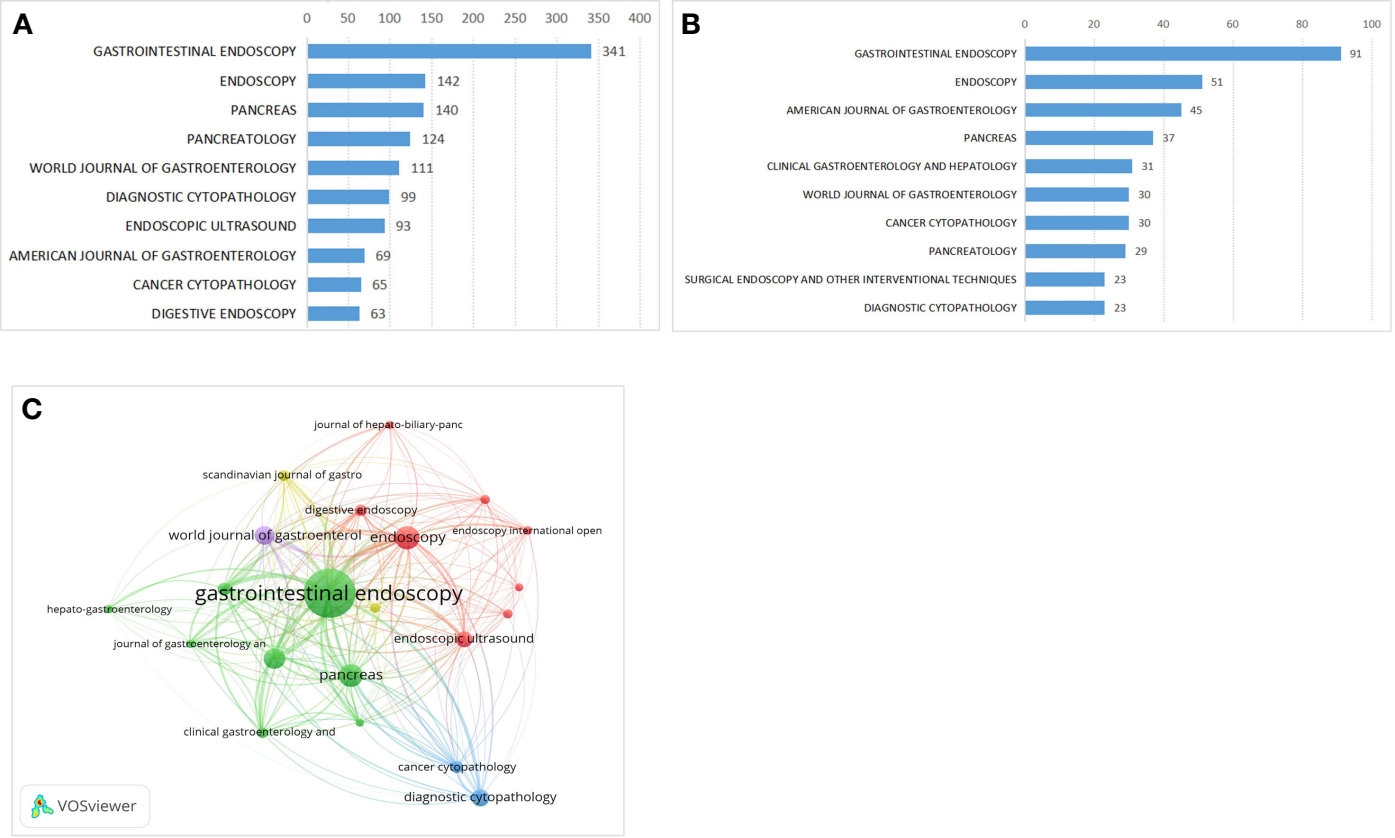
Journal distributions. **(A)** Top 10 journals of the publications, **(B)** Top 10 influential journals(H-Index). **(C)** The close relations between top 10 influential journals.

Until 2021, a total of 607 scholarly journals had published articles on this research. There are 92 journals with more than 10 publications, 24 journals with more than 30, and 10 journals with more than 60, 5 journals with more than 100. The top 10 journals with the greatest contribution to this research accounted for 30.63% (1247/4071) of the total publications included in this study (**Figure 5A**). Gastrointestinal Endoscopy (GIE, IF2021 = 9.427, Q1) was the most productive journal contributing 341 scientific publications in this field, followed by Endoscopy (n=142, IF2020 = 10.093, Q1), Pancreas (n=140, IF2020 = 3.327, Q3), Pancreatology (n=124, IF2020 = 3.996, Q3), World Journal of Gastroenterology (n=111, IF2020 = 5.742, Q3). Moreover, the top 10 most influential journals were listed based on the H-index (**Figure 5B**). GIE, Endoscopy, American journal of gastroenterology, Pancreas and Clinical gastroenterology, and hepatology ranked top 5, which means that these academic journals probably have a significant influence on this field. In **Figure 5C**, we showed the close relations between these journals. Larger bubbles for GIE, Endoscopy, and Pancreas indicated more publications in those fields. Additionally, Endoscopy, the World Journal of Gastroenterology, Pancreas, and Pancreatology all had active citation agreements with GIE.

### Author analysis

**Figure 6:**
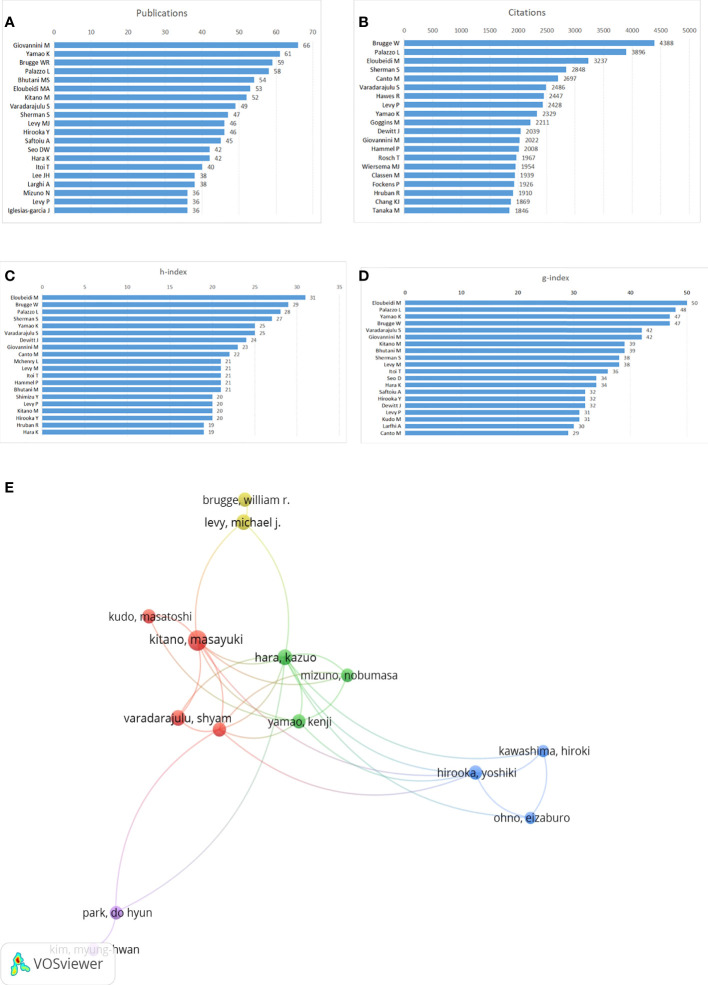
Author contributions. **(A)** Top 20 authors with the most publications, **(B)** Top 20 authors with the most ctiations, **(C)** h-index of publications from top 20 authors, **(D)** g-index of publications from top 20 authors, **(E)** Network map of co-authorship between authors with more than 30 publications.

According to the number of publications, influential authors were evaluated. A total of 260 authors had published at least 10 articles, 60 authors with more than 20 articles, and 7 authors with more than 50 articles. The author with the most articles published among these was Giovannini M (n=66), followed by Yamao K (n=61), Brugge WR (n=59), and Palazzo L (n=58) (**Figure 6A**). Moreover, the citation number, h-index, and g-index were also used to evaluate the author’s contributions. Citations in this field indicate that Brugge W ranked first (4388 citations), followed by Palazzo L (3896 citations) and Eloubeidi M (3237 citations) (**Figure 6B**).

The highest h-index was recorded by Eloubeidi M. (n=31), followed by publications from Brugge W (n=29) and Palazzo L (n=28) (**Figure 6C**). The publications’ g-index from Eloubeidi M (n=50) was also first, followed by that from Palazzo L. (n=48) and Yamao K (n=47) (**Figure 6D**). Collaborations between the authors were depicted in the network map in **Figure 6E**.

### Citation and co-citation of publications

**Figure 7:**
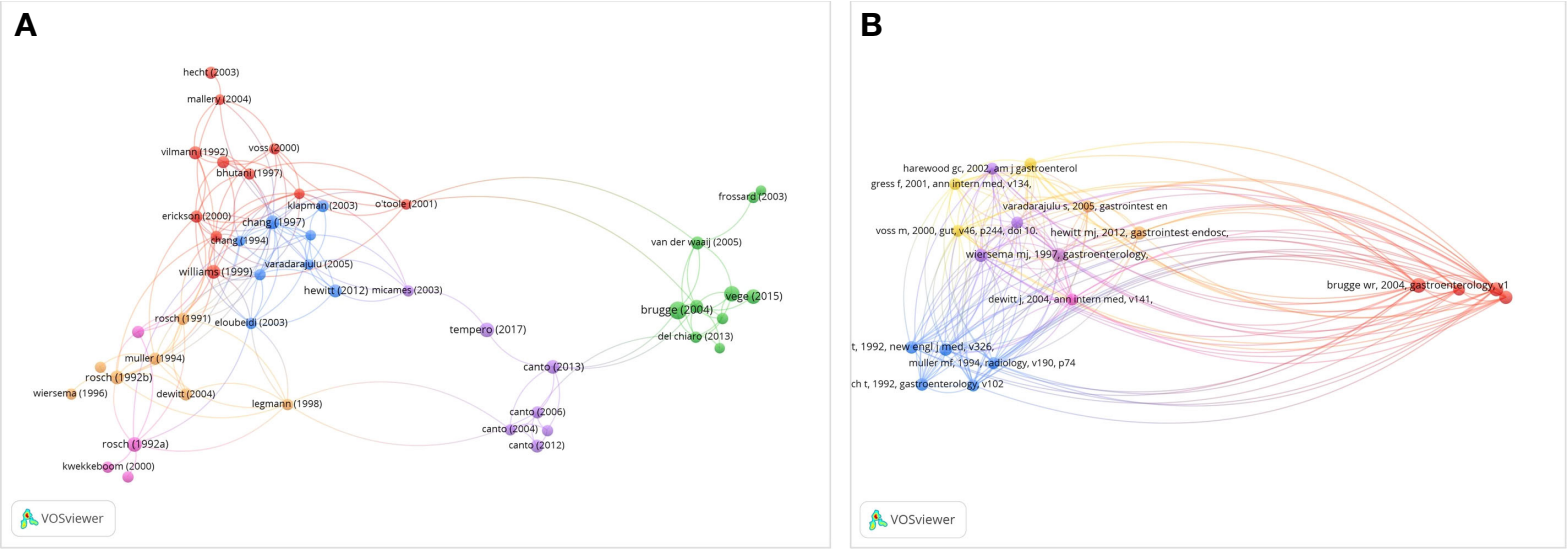
Network map of citation and co-citation publications. **(A)** Citation analysis of publications with more than 200 citations. **(B)** Co-citation analysis of references with more than 50 citations.

The citation network map of Publications that have more than 200 citations was shown in **Figure 7A**. And the top 10 articles with the highest citations were shown in **Table 1**. There were 939 citations for the publication written by Brugge W et al. from the Journal of Gastroenterology in 2004, followed by the article published by Tanaka M et al. in the Journal of Pancreatology in 2017, with 647 citations, and Rosch T et al. in the Journal of New England Journal of Medicine in 1992, with 532 citations. The citation network map of references that were co-cited in more than 150 citations is shown in **Figure 7B**. With active co-cited corporations with “Varadarajulu S” and “Dewitt J,” “Wiersema M” had the most publications among them all. In **Table 2**, the top 10 references with the most co-citations were presented. The top three references with the most citations were from Wiersema M [1997, 605 citations], Chang K [1997; 482 citations], and Brugge W [2004, 474 citations].

**Table 1:** Top 10 citation analysis of publications.

Rank	Author	Title	Source	Year	Country	Affiliation	Total Citation
1	Brugge W	Diagnosis of pancreatic cystic neoplasms: a report of the cooperative pancreatic cyst study	GASTROENTEROLOGY	2004	USA	Massachusetts General Hospital, Boston	934
2	Tanaka M	Revisions of international consensus Fukuoka guidelines for the management of IPMN of the pancreas	PANCREATOLOGY	2017	Japan	Shimonoseki City Hospital, Shimonoseki	647
3	Rosch T	Localization of pancreatic endocrine tumors by endoscopic ultrasonography	N ENGL J MED	1992	Germany	Technical University of Munich, Munich	532
4	Tempero M	Pancreatic Adenocarcinoma, Version 2.2017, NCCN Clinical Practice Guidelines in Oncology	J NATL COMPR CANCER NETW	2017	USA	University of California, San Francisco	529
5	Vege S	American gastroenterological association institute guideline on the diagnosis and management of asymptomatic neoplastic pancreatic cysts	GASTROENTEROLOGY	2015	USA	Mayo Clinic, Rochester	516
6	Williams D	Endoscopic ultrasound guided fine needle aspiration biopsy: a large single centre experience	GUT	1999	USA	Medical University of South Carolina, Charleston	484
7	Chang K	The clinical utility of endoscopic ultrasound-guided fine-needle aspiration in the diagnosis and staging of pancreatic carcinoma	GASTROINTEST ENDOSC	1997	USA	University of California, San Francisco	457
8	Canto M	International Cancer of the Pancreas Screening (CAPS) Consortium summit on the management of patients with increased risk for familial pancreatic cancer	GUT	2013	USA	Johns Hopkins University	443
9	Del Chiaro	European evidence-based guidelines on pancreatic cystic neoplasms	GUT	2018	USA	University of Colorado Cancer Center, Colorado	439
10	Hewitt M	EUS-guided FNA for diagnosis of solid pancreatic neoplasms: a meta-analysis	GASTROINTEST ENDOSC	2012	UK	Imperial College London, London	438

**Table 2:** Top 10 co-citation analysis of cited reference.

Rank	Author	Title	Source	Year	Country	Affiliation	Co-citations
1	Wiersema M	Endosonography-guided fine-needle aspiration biopsy: Diagnostic accuracy and complication assessment	GASTROENTEROLOGY	1997	USA	St Vincents Hosp, Indianapolis	605
2	Chang K	The clinical utility of endoscopic ultrasound-guided fine-needle aspiration in the diagnosis and staging of pancreatic carcinoma	GASTROINTEST ENDOSC	1997	USA	US Department of Veterans Affairs	482
3	Brugge W	Diagnosis of pancreatic cystic neoplasms: a report of the cooperative pancreatic cyst study	GASTROENTEROLOGY	2004	USA	Massachusetts General Hospital, Boston	474
4	Harewood G	Endosonography-guided fine needle aspiration biopsy in the evaluation of pancreatic masses	AM J GASTROENTEROL	2002	USA	Mayo Clinic, Rochester	461
5	Williams D	Endoscopic ultrasound guided fine needle aspiration biopsy: a large single centre experience	GUT	1999	USA	Medical University of South Carolina, Charleston	436
6	Rosch T	Endoscopic ultrasound in pancreatic tumor diagnosis	GASTROINTEST ENDOSC	1991	Germany	Technical University of Munich, Munich	433
7	Voss M	Value of endoscopic ultrasound guided fine needle aspiration biopsy in the diagnosis of solid pancreatic masses	GUT	2000	France	Beaujon Hospital, Clichy	425
8	Gress F	Endoscopic ultrasonography-guided fine-needle aspiration biopsy of suspected pancreatic cancer	ANN INTERN MED	2001	USA	Winthrop-University Hospital,Mineola	413
9	Eloubeidi M	Endoscopic ultrasound-guided fine needle aspiration biopsy of patients with suspected pancreatic cancer: Diagnostic accuracy and acute and 30-day complications	AM J GASTROENTEROL	2003	USA	Univ Alabama Birmingham,Birmingham	381
10	Palazzo L	Endoscopic ultrasonography in the diagnosis and staging of pancreatic adenocarcinoma. Results of a prospective study with comparison to ultrasonography and CT scan	ENDOSCOPY	1993	France	Beaujon Hospital, Clichy	359

### Analysis of keywords

**Figure 8:**
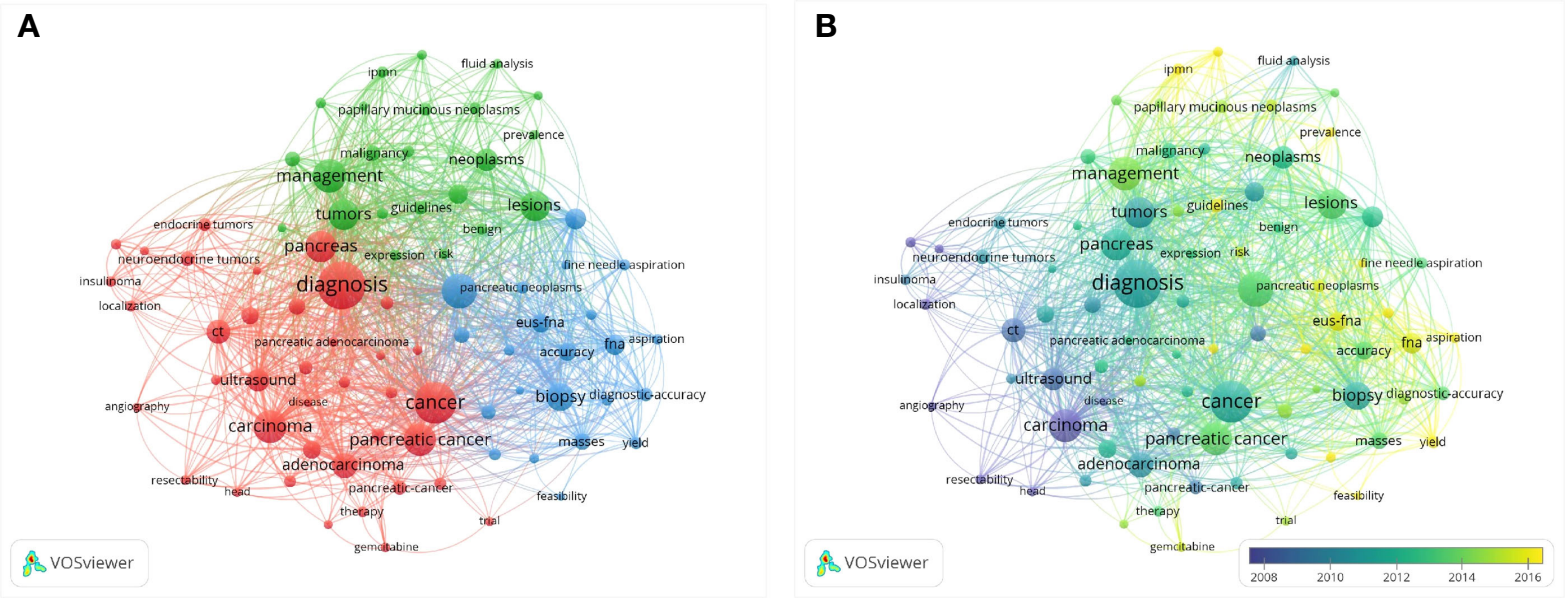
Co-occurrence analysis of keywords. **(A)** Network visualization map of keywords, **(B)** Overlay visualization map of keywords.

We extracted keywords from the publications and analyzed co-occurrence *via* VOSviewer. A co-occurrence association was created between two keywords when they occurred in the same article. Strong co-occurring keywords can more precisely reveal research hotspots than a single term. A total of 100 keywords that were found to appear more than 50 times were used to construct the network visualization map (**Figure 8A**). And the top 10 keywords were diagnosis, cancer, management, pancreatic cancer, carcinoma, biopsy, tumors, pancreas, adenocarcinoma, lesions. The line that connects two terms in this keyword network map gets changed off as a sign. The number of occurrences was represented by the size of the bubble. The network visualization map’s colors show the several clusters that the keywords formed. And in **Figure 8B**, each keyword was colored according to the moment when it emerged. The color of keywords served as a visual representation of when they first appeared, with blue implying an early introduction and yellow suggesting a more recent emergence.

## Discussion

### Research trends

A bibliometric analysis was performed to introduce the development of EUS in pancreatic tumor diseases based on articles published from 1984 to 2021. In 1984, there was only one publication, which might because the area was still developing, but increased to 310 in 2020, especially in the last decade (2010-2021, the number of annual publications has exceeded 160), which reflects the hot research status in this field in recent years. Our investigation revealed a general upward trend in the annual production of this research, indicating that more people have been paying attention to it lately. The top five producing countries were the USA, Japan, Germany, France, and Italy, demonstrating their prominence in the field of this research. According to the country collaboration map, North America and Europe have produced the most articles among the top 20 nations, indicating that these two areas may have great potential for study and growth. These top contributing nations and regions were connected on the network map and nation collaboration map, demonstrating the research’s global scope. This sort of international collaboration might promote academic exchange, draw better scientists to the topic, and speed up research on the application of EUS in pancreatic tumors.

Author analyses allow for a more thorough and unbiased assessment of researchers’ contributions, the quality of their work, and their academic standing in a field of study. The 20 most productive authors in this area were also active scholars. Brugge W has the most citations, Eloubeidi M currently has the greatest h-index, and Giovannini M published the most publications. According to our study, the most well-known and respected authors in the field using these facts and indications may be identified. Notably, the majority of the authors were from organizations affiliated with universities and hospitals. Additionally, we evaluated the co-authorship of more than 20 papers and discovered that while there was some coordination and contact, it wasn’t close. Therefore, author collaboration should be the main topic of future study. To put it another way, by identifying these leaders, we would be able to review the literature before starting new EUS-related research and quickly and accurately understand how this subject has developed.

As to the journals, the results showed that GIE, Endoscopy, Pancreas, Pancreatology, and World Journal of Gastroenterology were the most prevalent journals. The journals shown in the network map’s co-citation frequency were closely related to one another in the field. This could guide new researchers who want to conduct their research and submit remarkable findings in this area. These journals mainly focus on clinical studies, and this research will mainly focus on the clinical field in the future.

### Research status

The number of citations that an article obtains may be the most crucial bibliometric characteristic because it indicates how relevant and significant a study is in academic research. In this study, we analyzed the publications with citations from 1984 to 2021. And found that among the 4071 publications, 272 (6.69%) articles were cited more than 100 times. And the top 10 cited articles were listed in detail. Overall, eight of the top 10 publications were related to the diagnosis of EUS in pancreatic diseases, which indicated the vital function of EUS in the diagnosis of pancreatic diseases. According to keywords co-occurrence analysis, hotspots, subjects and trends are able to identify to guide researchers or clinicians to comprehend the development of related studies of EUS in pancreatic tumor diseases. In **Figure 8**, as was shown in the network map, the co-occurrences of keywords were analyzed and mainly clustered into three research subjects or trends. In this study, the hotpots of the progress of EUS in pancreatic diseases were as follows.

### Classification of pancreatic tumor disease

Pancreatic tumor diseases were classified into benign tumors and malignant tumors. And benign included serous cystadenoma (SCN), pancreatic intraepithelial neoplasia (PanIN), mucinous cystic neoplasm (MCN), intraductal papillary mucinous neoplasm (IPMN), intraductal oncocytic papillary neoplasm (IOPN), intraductal tubulepapillary neoplasm (ITPN), and pancreatic neuroendocrine neoplasms(pNENs) (11). Among malignant tumors, pancreatic ductal adenocarcinoma (PDAC) has the highest incidence and malignancy, others included acinic cell carcinoma, pancreatic blastoma, and so on. Notably, endoscopic ultrasonography is crucial for the detection and management of both benign and malignant pancreatic tumors (12).

### Development of EUS in the diagnosis of pancreatic tumor diseases

EUS can directly display the pancreatic tissue structure and the adjacent relationship of the surrounding organs. Compared with traditional imaging examination, EUS has a higher sensitivity and specificity in the diagnosis of pancreatic diseases and was recommended in guidelines. According to our study, the development of EUS in the diagnosis of pancreatic diseases were shown in detail.

In 1980, DiMagno. et al. (2) developed EUS for the evaluation of digestive diseases for the first time. As the whole pancreas can be visualized without any blind spots in the ultrasonographic images, EUS had gradually become an important diagnostic tool for pancreatic diseases. For the next decades, EUS was primarily used to aid in the diagnosis of pancreatic diseases. Strohm WD et al. (13) applied it to diagnose a pancreatic tumor in 1984 for the first time. However, on account of the lack of pathological confirmation of EUS, the clinical role of EUS had been restricted. But the advent of EUS-guided fine needle aspiration (EUS-FNA) offered a new diagnostic strategy for pancreatic tumor disease. In 1992, Peter et al. (14) successfully performed EUS-FNA on a patient with a pancreatic disease which could not be diagnosed by conventional methods, and obtained pancreatic tissue, which was suggested by pathology as mucinous cystic neoplasm. Pathological diagnosis, which cannot be obtained by EUS but could be acquired by EUS-FNA, is the gold standard for many diseases, which has promoted the development of EUS and made great progress. The method of EUS-FNA is not limited to pancreatic diseases, but applies to other lesions of the adjacent organs and structures.

In 1995, Kato et al. (15) in Japan first used the perfusion imaging approach in combination with EUS, an enhanced harmonic EUS has been developed, which was performed by injecting carbon dioxide through the angiographic catheter inserted into the celiac trunk or superior mesenteric artery during EUS circumferential scanning. Because of its advantages of high-resolution ultrasound and contrast-enhanced ultrasound, it is of great significance for distinguishing the characterization of solid and cystic pancreatic lesions, judging the stage of pancreatic cancer and vascular involvement (16). In 2002, Maurits J et al. (17) reported EUS-guided needle biopsy (EUS-TNB), which is similar to EUS-FNA but can obtain more reliable tissue for pathological diagnosis. In 2007, Janssen J et al. (EUS-EG 2007) reported the clinical use of EUS-elastography (EUS-EG) in pancreatic disease. EG is the technology that can image the differences of distortion between the soft tissue and the hard tissue in real-time. So it can study imaging of tumors and spreading diseases that cannot be detected by traditional ultrasound. Since then, EUS-EG has been widely used in the differential diagnosis of pancreatic solid mass, benign and malignant lymph nodes, gastrointestinal submucosal masses, and some other solid tumors. Endoscopic confocal laser endomicroscopy (CLE) is a new endoscopic confocal laser microscope that integrates traditional endoscopy under microscopic imaging technology, real-time histologic diagnosis in the endoscopic examination at the same time, through dynamic observation on the surface of the magnified 1000 times of particular organization cells, blood vessels, basement membrane, and stroma morphology and structure, and was called ‘optical biopsy under endoscopy’ (18). In 2011, Konda et al. (19) first applied this technique to evaluate the feasibility of nCLE of pancreatic lesions. Shortly afterward, the technique gradually matured and is mainly used in the differential diagnosis of pancreatic cystic lesions, solid pancreatic mass and enlarged lymph nodes.

Endoscopic ultrasonography (EUS) can perform personalized and real-time scanning of pancreatic lesions in the stomach or duodenal cavity at the nearest distance, and is one of the most sensitive methods for finding small pancreatic lesions. It can find lesions only 2mm in size, which has become one of the most accurate methods for locating pancreatic cancer and is conducive to the early detection of pancreatic cancer. Since its inception in the 1980s, this technique has played a huge role in the diagnosis of pancreatic diseases, especially with the development of assistive imaging, such as the CHE, EG, CLE and so on, and the development of EUS-FNA and EUS-FNB has brought the role of EUS to a new level. At present, EUS has been recommended as a critical diagnostic way in many clinical guidelines.

### Development of EUS in the treatment of pancreatic tumor diseases

EUS not only can be used as a diagnostic tool for pancreatic diseases, but also has made significant breakthroughs in the transformation into minimally invasive intervention therapy with the continuous development of technological progress, and has become an important treatment method for pancreatic diseases.

EUS-guided ablation is a reliable treatment for patients with inoperable pancreatic diseases, high surgical risk, or rejection of surgery, mainly including ethanol ablation, mixed cryogenic ablation, radiofrequency ablation, photodynamic ablation, and laser ablation. EUS- radiofrequency ablation (RFA) is a novel tumor treatment method, which causes coagulation necrosis in surrounding tissues by releasing heat through a high-frequency current, and is widely used in the treatment of liver, lung and kidney tumors. Goldberg et al. (20) in 1999 reported the use of EUS-guided radiofrequency ablation for pancreatic diseases in porcine models for the first time. This study indicated that EUS-guided radiofrequency ablation can be used to produce coagulation necrosis in the porcine pancreas lesions. As technology continues to advance, it has become the most widely used way of ablation in the pancreas at present.

EUS-guided photodynamic ablation (PDT) is a tumor-specific ablative therapy that combines photosensitive drugs with EUS-guided light irradiation to produce oxygen-free radicals leading to cell death. Chan et al. (21) in 2003 first introduced the application of EUS-guided photodynamic therapy (PDT) in the pancreas. This study confirmed that EUS-PDT is safe and effective for advanced focal pancreatic cancer to a certain extent, but it remains to be further proved by further studies.

Pain is the most common complication in patients with pancreatic cancer, and peritoneal plexus block is a first-line adjuvant for the treatment of pain in patients with pancreatic cancer. Wiersema et al. (22) proposed in 1996 that EUS-celiac plexus neurolysis (CPN) could relieve the pain of patients with pancreatic patients. In addition, EUS-CPN can also improve the survival rate of patients with pancreatic cancer. Fuji-Lau et al. (23) showed in a case-control study that patients treated with EUS-CPN had a longer survival time compared with patients without. However, EUS-CPN does not eliminate pain, but only relieves it to some extent.

EUS-guided intratumoral fine-needle injection (EUS-FNI) is a relatively new targeted therapy that aims to maximize intratumoral drug concentration while minimizing systemic exposure and drug toxicity. As a result, it can be applied as a preoperative intervention to reduce tumor size or as a palliative care measure for unresectable tumors with obstructive symptoms. Currently, EUS-FNI can be used for a variety of interventions, including chemotherapy, immunotherapy, oncolytic virus therapy and tumor implantation. Chang et al. (24) in 2000 conducted the first clinical trial using EUS-FNI to directly inject anti-tumor agents into local cancer, which delivered allogeneic mixed lymphocyte cultures to unresectable pancreatic adenocarcinoma and the median survival of patients was 13.2 months with no operation-related complications. EUS-guided ethanol ablation is a novel alternative therapy for pancreatic cystic tumors and pancreatic neuroendocrine tumors in recent years, and its safety and feasibility have been reported in some studies (25, 26). EUS-FNI has precise localization and fewer adverse reactions, which can improve the clinical therapeutic effect of pancreatic masses.

Peripancreatic effusion is a common local complication after pancreatitis or pancreatic trauma. The fluid is divided into acute peripancreatic fluid accumulation, acute necrotic accumulation, pancreatic pseudocyst and walled-off necrosis (27). Pancreatic pseudocyst has clear cyst walls and few necrotic components, which usually appear 4 weeks after acute pancreatitis or pancreatic trauma, and a small part of them are secondary to chronic pancreatitis (28). The most common decompression indications of pancreatic pseudocyst are abdominal pain, infection, and biliary obstruction, and cysts (> 6cm in diameter) that continue to grow or remain unhealed for more than 6 weeks. Pancreatic abscesses and walled-off necrosis often need drainage intervention. Drainage is generally performed 4-6 weeks after the formation of peripancreatic effusion and full liquefaction of mature necrotic material in the capsule wall (29). In 1992, Grimm et al. (30) first proposed EUS-guided drainage therapy for pancreatic pseudocysts., which concluded that EUS-guided drainage is as effective as percutaneous drainage in the treatment of pancreatic pseudocysts.

Interstitial brachytherapy is an effective method for local control of pancreatic malignancies. Radioactive particles are implanted in and around the target tissue, exposing the target tissue to γ rays, resulting in local tissue damage and tumor ablation. Importantly, these radioactive seeds have a very low dose rate and penetration depth of no more than 1.7 cm, thus minimizing radiation exposure and damage to adjacent organs (31). Sun et al. (32) in 2005 showed for the first time in a pig model that the implantation of radioactive particles into pancreatic tissues guided by EUS is a safe and feasible brachytherapy method. The most popular radioactive particle is iodine-125, which has a half-life of 59.7 days and is suitable for fast-growing tumors such as pancreatic cancer.

### Strengths and limitations

An objective quantitative analysis was made of the existing literatures by using bibliometrics. Researchers and clinicians’ understanding of the worldwide presentation and patterns of EUS development in pancreatic tumors may be improved by these findings and recommendations. Our research has several advantages. First of all, this study for the first time used the bibliometrics analysis method with a deep insight to present the global development, status and trend of research on EUS in pancreatic tumor diseases. Secondly, our study uses widely used tools, which ensure the reliability of the data. Thirdly, bibliometric analysis is more thorough and objective than conventional literature evaluations. However, similar to other bibliometric analyses, this study also has some limitations. First, we only searched articles in the WOS database; we did not search other databases like PubMed or Embase, thus some publications may have been missed. However, it is worth noting that the WOC is the one that is most frequently utilized in the bibliometric analysis. Secondly, there may be differences between bibliometric analysis results and actual research conditions. Our conclusions are based on research that has been published, however some crucial material might not have appeared in academic papers. Thirdly, there may not have been a thorough discussion in our research because some of the recently released significant articles may not have received enough attention from scholars. Despite these limitations, our study provides an elaborate global perspective on EUS of pancreatic tumor diseases research over the past five decades.

## Conclusion

A thorough overview of the global research on EUS in pancreatic tumor diseases during the past decades was shown using bibliometric analysis. This topic is going through a period of tremendous development, and more scholars are becoming interested in it. Research results indicate that the USA contributed the most to productivity, and it connected closely with other countries, such as Japan, China, England, Germany, France, Italy, and Japan. And journals such as GIE, Endoscopy, Pancreas, Pancreatology and World Journal of Gastroenterology pay close attention to developments in the field. With the development of endoscopic technology and equipment, EUS has gradually developed from a simple diagnostic method to a significant part of interventional treatment of digestive system diseases, especially playing an important role in the diagnosis and treatment of pancreatic diseases. The topic of EUS in Pancreatic tumor diseases is worthy of continued follow-up by researchers, and we believe that this study contributes valuable information for researchers and clinicians.

## Data availability statement

The original contributions presented in the study are included in the article/supplementary material. Further inquiries can be directed to the corresponding author.

## Author contribution

CX: data collection and paper writing. HY: statistical analysis. KZ: interpretation of data. ZW and XY: literature search. HH: manuscript review. All authors contributed to the article and approved the submitted version.

## Funding

Funded by the National Outstanding Youth Science Fund Project of National Natural Science Foundation of China (to H.J.H.), No. 82022008.

## Conflict of interest

The authors declare that the research was conducted in the absence of any commercial or financial relationships that could be construed as a potential conflict of interest.

## Publisher’s note

All claims expressed in this article are solely those of the authors and do not necessarily represent those of their affiliated organizations, or those of the publisher, the editors and the reviewers. Any product that may be evaluated in this article, or claim that may be made by its manufacturer, is not guaranteed or endorsed by the publisher.
